# Sanguinarine Alleviates the Adverse Effects of Excessive Dietary Histamine on Growth and Intestinal and Hepatic Health in Juvenile American Eels (*Anguilla rostrata*)

**DOI:** 10.3390/ani16101556

**Published:** 2026-05-20

**Authors:** Yichuang Xu, Runan Chen, Xinyu Hu, Yuqin Yan, Jinyue Yang, Shaowei Zhai

**Affiliations:** 1 Fisheries College, Jimei University, Xiamen 361021, China; xuyichuang@jmu.edu.cn (Y.X.); raynachen054@163.com (R.C.); 202411908017@jmu.edu.cn (X.H.); 202511710015@jmu.edu.cn (Y.Y.); 2 Engineering Research Center of the Modern Technology for Eel Industry, Ministry of Education, Xiamen 361021, China; 3 Qingdao Conson Oceantec Valley Development Co., Ltd., Qingdao 266003, China; 4 College of Ocean Food and Biological Engineering, Jimei University, Xiamen 361021, China; 202461000113@jmu.edu.cn

**Keywords:** sanguinarine, histamine, growth performance, liver, intestine

## Abstract

Histamine is a significant risk factor in brown fishmeal and negatively affects histamine-sensitive fish species. The present study aimed to explore the potential protective effects of dietary sanguinarine supplementation against the adverse impacts of excessive dietary histamine on growth and intestinal and hepatic health in American eels. Four experimental diets were formulated: the basal diet (control diet), the basal diet supplemented with 500 mg kg^−1^ of histamine (HH diet), and the HH diet supplemented with 100 mg kg^−1^ and 200 mg kg^−1^ of sanguinarine (HH+SAN100 and HH+SAN200 diets, respectively). After a 10-week feeding trial, both sanguinarine supplementation diets alleviated HH-diet-induced growth retardation, reduced feed utilization, serum dyslipidemia, and hepatic and intestinal structural damage; decreased hepatic and intestinal antioxidant capacity; increased intestinal and hepatic malondialdehyde levels; inhibited intestinal lipase activities; elevated intestinal pro-inflammatory-related gene abundance; and lowered intestinal anti-inflammatory-related gene expression. Furthermore, the HH+SAN100 diet partially ameliorated HH-diet-induced gut microbiota dysbiosis and intestinal metabolic disturbances. Collectively, these findings identify sanguinarine as a functional dietary additive that mitigates histamine-associated growth suppression and intestinal and hepatic injury in American eels, promoting the sustainable utilization of brown fishmeal in American eel culture.

## Simple Summary

Brown fishmeal is an essential protein source in aquafeeds. However, histamine commonly accumulates in brown fishmeal and is an important dietary toxicant in aquafeeds, thereby limiting the safe utilization of brown fishmeal in feeds for histamine-sensitive fish species. Sanguinarine is recognized for its anti-inflammatory, hepatoprotective, and enteroprotective activities. This study demonstrated that dietary sanguinarine supplementation alleviated high-histamine-diet-induced growth retardation, reduced feed utilization, serum dyslipidemia, and hepatic and intestinal damage; and inhibited intestinal lipase activities, intestinal inflammatory response, gut microbiota dysbiosis, and intestinal metabolic disturbances of American eels (*Anguilla rostrata*). Consequently, these findings indicate that sanguinarine is a practical additive to reduce high-histamine-diet-induced health risks in American eels.

## 1. Introduction

The presence of anti-nutritional factors, environmental contaminants, and naturally occurring toxicants in aquafeeds constitutes a critical hindrance for sustainable aquaculture, severely compromising the health and growth performance of cultured fishes [1,2,3]. Among these factors, histamine is of particular concern because it commonly accumulates in brown fishmeal and represents an important dietary toxicant in aquafeeds [4]. Histamine is mainly formed through bacterial decarboxylation of free L–histidine during raw-material spoilage and improper storage, and elevated histamine levels are commonly detected in high-protein ingredients that stored under unsuitable conditions or contaminated with microorganisms [3,4]. It was reported that histamine levels in some low-quality brown fishmeal even surpass 1000 mg kg^−1^ [4]. Upon intestinal absorption, excessive dietary histamine enters the systemic circulation and exerts toxicity [5]. While it primarily triggers structural injury, oxidative damage, and microbiota dysbiosis within the intestine, it concurrently precipitates hepatic degeneration, immunosuppression, and muscle sclerosis, ultimately resulting in severe feed inefficiency and growth retardation in some fish species [3,6,7,8,9]. Given this constraint, effective nutritional strategies are needed to reduce histamine-associated damage and improve the safe use of brown fishmeal.

In recent years, plant-derived functional additives with antioxidant and anti-inflammatory properties have attracted increasing attention as dietary interventions in aquaculture for mitigating toxicant-induced damage [10,11]. Sanguinarine, a major bioactive benzophenanthridine alkaloid extracted from *Macleaya cordata*, has been reported to possess broad-spectrum antimicrobial, anti-inflammatory, hepatoprotective, and enteroprotective activities [12,13]. A recent study has demonstrated that dietary sanguinarine supplementation effectively inhibited the upregulation of intestinal pro-inflammatory cytokines caused by high level cottonseed and rapeseed meal diets in grass carp (*Ctenopharyngodon idella*) [14]. Additionally, dietary sanguinarine supplementation was shown to protect the liver of grass carp from high-fat-diet-induced oxidative damage [15]. Shi et al. [13] further found that dietary sanguinarine supplementation attenuated hydrogen-peroxide-induced inflammation and oxidative stress in liver of rice field eel (*Monopterus albus*). Sanguinarine has also been shown to protect channel catfish (*Ictalurus punctatus*) against pathogenic infection [16]. Although sanguinarine has been shown to alleviate different forms of stress and improve the health of the intestine and liver, whether it can counteract the systemic toxicity and growth inhibition induced by excessive dietary histamine remains unknown. Mechanistically, while the general antioxidant and anti-inflammatory properties of sanguinarine are well documented [13,14,15], its specific interaction with histamine stress requires further elucidation. Therefore, we hypothesized that sanguinarine mitigated histamine-induced tissue injury not merely through general ROS scavenging but specifically by inhibiting the NF-κB signaling pathway and suppressing downstream pro-inflammatory cytokines. Addressing this gap may provide a practical nutritional strategy for improving the safe utilization of brown fishmeal in aquafeeds.

Eels (*Anguilla* spp.) are globally recognized for their high nutritional value and premium economic status. Among them, the American eel (*Anguilla rostrata*) is the prominent farmed eel species worldwide. Due to their carnivorous nature, American eels have a high dietary protein requirement, and premium white fishmeal has traditionally served as the primary protein source in formulated feeds [3,17]. Recently, the rising cost and limited supply of white fishmeal have driven the utilization of brown fishmeal in American eel diets [17]. However, incorporating elevated proportions of brown fishmeal into diets inevitably leads to a high dietary histamine content [17]. Our previous studies demonstrated that dietary histamine exceeding 355.31 mg kg^−1^ not only suppressed growth but also damaged the intestinal and hepatic health of American eels [3,18]. Therefore, developing effective nutritional strategies to mitigate histamine toxicity is essential for the safe and broader use of brown fishmeal in American eel feeds. Accordingly, the present research was carried out to assess the impact of dietary supplementation with 100 or 200 mg kg^−1^ of sanguinarine on the growth performance and intestinal and hepatic health of American eels fed with a high-level of histamine, which may provide a practical strategy to improve brown fishmeal utilization and reduce feed costs in American eel farming.

## 2. Materials and Methods

### 2.1. Experimental Design and Diet Formulation

A commercial formulated diet was used as the basal diet, primarily comprising white fishmeal, brown fishmeal, α–starch, extruded soybean, and vitamin and mineral premixes (Table 1). Proximate analysis showed that the basal diet contained 47.87% of crude protein, 5.17% of crude lipid, 12.98% of crude ash, and 6.22% of moisture. Based on our previous findings [3,18], a high-histamine (HH) diet was prepared by supplementing the basal diet with 500 mg kg^−1^ of histamine (#S20188, 98% purity; Shanghai Yuanye Bio-Technology Co., Ltd., Shanghai, China). The analyzed histamine concentrations in the basal and HH diets were 124 and 624 mg kg^−1^, respectively. Additionally, the HH diet was further supplemented with 100 or 200 mg kg^−1^ of sanguinarine to produce the HH+SAN100 and HH+SAN200 diets, respectively. This sanguinarine product, standardized to an effective content of 3.75%, was supplied by Hunan Micolta Bioresource Inc. (Changsha, China).

### 2.2. Feeding and Sampling

American eels were sourced from a commercial fish farm in Fujian Province, China. Before the feeding trial, American eels were acclimated for four weeks and fed a commercial diet to apparent satiation twice daily. After acclimation, 400 uniformly sized American eels (initial body weight: 16.00 ± 0.06 g fish^−1^, means ± S.D.) were randomly allocated into 16 cylindrical tanks, with 25 fish per tank and four replicates per treatment. The feeding trial lasted 10 weeks. American eels were fed to satiation twice daily at 6:00 and 18:00 during the feeding trial. Following a feeding period of 30 min, the uneaten feed was removed and dried to record daily feed consumption. Throughout the experiment, water quality parameters were monitored daily and maintained within the following ranges: temperature, 24–26 °C; pH, 7.0–7.8; dissolved oxygen, 8.0–9.5 mg L^−1^; total ammonia nitrogen, <0.20 mg L^−1^; and nitrite, 0.02–0.06 mg L^−1^.

At the end of the feeding experiment, the eels were fasted for 12 h before sampling. Subsequently, 18 fish were randomly selected from each tank and anesthetized with 100 mg L^−1^ of eugenol solution. Blood samples were collected from nine individuals per tank to analyze serum biochemical parameters. Intestinal and hepatic samples were immediately frozen in liquid nitrogen and stored at −80 °C until further analysis.

### 2.3. Analytical Methods

#### 2.3.1. Determination Growth Performance and Feed Utilization

After the completion of the feeding trial, the fish in each tank underwent a 12-h starvation period before being weighed to assess their growth performance. The parameters were calculated using the following equations:

Weight gain rate (WGR, %) = 100 × (FBW − IBW)/IBW,

where IBW and FBW refer to initial body weight and final body weight, respectively.

Specific growth rate (SGR, % d^−1^) = (Ln FBW − Ln IBW)/trial days (d) × 100;

Feed intake (FI, g fish^−1^) = feed intake of each tank (g)/fish number per tank;

Feed efficiency (FE, %) = 100 × (FBW − IBW)/feed intake per tank (g);

Survival rate (SR, %) = (final fish number per tank)/(initial fish number per tank) × 100.

#### 2.3.2. Serum Biochemical Parameters

The levels of albumin (ALB, #A028-1-1), high-density lipoprotein cholesterol (HDL-C, #A112-1-1), low-density lipoprotein cholesterol (LDL-C, #A113-1-1), total cholesterol (TC, #A111-1-1), triglyceride (TG, #A110-1-1), and D-lactate (D-lac, #A019-3-1), along with the activities of diamine oxidase (DAO, #A088-2-1), glutamic-oxalacetic transaminase (GOT, #C010-1-1), and glutamic–pyruvic transaminase (GPT, #C009-1-1), were measured using commercial kits (Nanjing Jiancheng Bioengineering Co., Ltd., Nanjing, China), following the manufacturer’s instructions.

#### 2.3.3. Histological and Ultrastructural Observation

Hematoxylin and eosin (H&E) staining was carried out according to our previous study [18]. Briefly, the intestinal samples were first fixed in 4% paraformaldehyde for 24 h at room temperature and subsequently dehydrated through a graded ethanol series. The dehydrated samples were embedded in paraffin, and serial sections with a thickness of 5 μm were prepared. The sections were mounted on glass slides, deparaffinized in xylene, and rehydrated through descending concentrations of ethanol. Subsequently, the sections were stained with hematoxylin for 3 min and eosin for 1 min, followed by washing with distilled water. After dehydration and sealing with neutral resin, the stained sections were observed under a light microscope and the images were analyzed utilizing Image-Pro Plus 6.0 software.

For scanning electron microscope (SEM) observation, intestinal samples were cut into small pieces and immediately fixed in 2.5% glutaraldehyde at 4 °C for 24 h. After fixation, the samples were rinsed three times with phosphate-buffered saline and dehydrated through a graded ethanol series. The dehydrated samples were further dried using a critical point dryer, mounted onto aluminum stubs, and sputter-coated with a thin layer of gold. Finally, the prepared samples were observed and photographed utilizing a scanning electron microscope.

#### 2.3.4. Determination the Antioxidant Capacity

Approximately 0.5 g of intestinal tissue were collected from each tank and homogenized in pre-cooled physiological saline at a ratio of 1:9 (*w*/*v*) using a tissue grinder. The homogenates were centrifuged at 3000 rpm for 10 min at 4 °C, and the resulting supernatants were collected for subsequent analysis. The activities of catalase (CAT, #A007-1-1), glutathione peroxidase (GSH-Px, #A005-1-2), and superoxide dismutase (SOD, #A001-1-1) activities, along with the total antioxidant capacity (T-AOC, #A015-2-1) and the level of malondialdehyde (MDA, #A003-1-2), were measured using the commercial kits (Jiancheng Biotech Co., Ltd., Nanjing, China).

#### 2.3.5. Assessment of Intestinal Digestive Enzymes

The preparation of tissue homogenates followed the protocol described in Section 2.3.4. The activities of digestive enzymes, including amylase (#C016-1-1) and lipase (#A054-2-1), were analyzed with the kits (Nanjing Jiancheng Bioengineering Co., Ltd.). The activity of protease was assessed using Folin phenol reagent.

#### 2.3.6. Real-Time Quantitative PCR (RT-PCR)

Total RNA was extracted from the intestinal tissues using a commercial RNA extraction kit (#RC112, Vazyme Biotech Co., Ltd., Nanjing, China). RNA concentration was measured using a spectrophotometer (NanoDrop One, Thermo Fisher Scientific Inc., Waltham, MA, USA) and the purity was determined by 260/280 and 260/230 absorbance ratios. For complementary DNA (cDNA) synthesis, 1.5 μg of total RNA from each sample was reverse transcribed. The reverse transcription reaction was performed at 37 °C for 15 min, followed by enzyme inactivation at 85 °C for 15 s, and the synthesized cDNA was stored at −20 °C until use. The reaction mixture contained a cDNA template, forward and reverse primers, the SYBR Green qPCR Master Mix, and nuclease-free water in a final volume according to the kit instructions (#Q711-02, Vazyme Biotech Co., Ltd., Nanjing, China). The amplification procedure of RT-PCR consisted of an initial denaturation step followed by 40 cycles of denaturation, annealing, and extension. The relative abundance of *tnf-α*, *ifn-γ*, *il-10*, *tgf-β*, and *nf-κb* were normalized to *gapdh* and calculated using the 2^−ΔΔCT^ method. The amplification primers are shown in Table 2.

#### 2.3.7. Analysis of Gut Microbiota

The characterization of the intestinal microbiota was conducted following our previous studies [18,19]. Gut microbial diversity was assessed by high-throughput sequencing of the 16S rDNA V3–V4 region on the Illumina MiSeq PE300 platform. After sequencing, raw reads were assembled, quality-filtered, and checked for chimeras using QIIME (v1.8.0) to obtain high-quality sequences, which were then clustered into OTUs and taxonomically annotated. Alpha-diversity indices were calculated in Mothur (v1.31.2) with reference to the SILVA (v138) database.

#### 2.3.8. Metabolomics Analysis

Tissue samples were homogenized and sonicated in extraction solvent (methanol:acetonitrile:water = 2:2:1, *v*/*v*/*v*) containing an internal standard mixture, incubated at −40 °C for 1 h, and centrifuged (12,000 rpm, 15 min, 4 °C). Equal aliquots of the supernatants were pooled to generate quality control (QC) samples. Metabolomic profiling was conducted with UPLC–MS/MS (Thermo Fisher Scientific) using an ACQUITY UPLC BEH Amide column, with pooled QC samples prepared by mixing equal aliquots of all supernatants. Raw data were converted to mzXML format via ProteoWizard (v3.0.10827), followed by peak extraction, alignment, and integration using XCMS. Metabolite annotation was performed by matching against an in-house MS2 database using R packages (v4.0.0).

### 2.4. Statistical Analysis

The data were expressed as means ± standard deviation (S.D.). (*n* = 4). Statistical analysis was carried out using SPSS 17.0 software. Student’s *t*-test was employed to compare the discrepancies between the two groups. The Kruskal–Wallis (KW) test, a non-parametric method, was applied for the analysis of differential bacteria in the intestine. Statistically significant differences were determined at *p* < 0.05.

## 3. Results

### 3.1. Growth Performance and Feed Utilization

**Figure 1:**
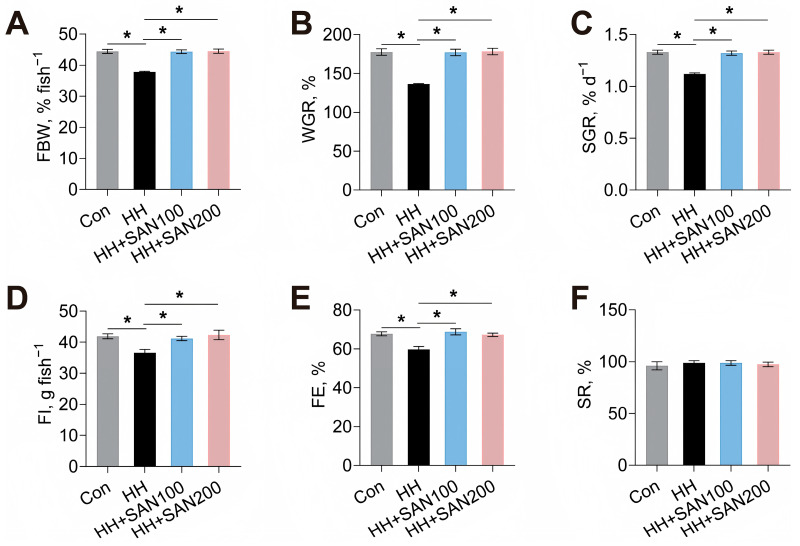
Effects of dietary histamine and sanguinarine on FBW (**A**), WGR (**B**), SGR (**C**), FI (**D**), FE (**E**), and SR (**F**) of juvenile American eels. Values are shown as mean ± S.D. (*n* = 4). Results were analyzed using independent *t*-test, * *p* < 0.05. Con, the control diet; HH, the high-histamine diet; HH+SAN 100, the HH+SAN100 diet; HH+SAN200, the HH+SAN200 diet.

Firstly, the effects of dietary sanguinarine and histamine on the growth performance and feed utilization of American eels were assessed (Figure 1). The present results revealed that compared to the control diet, the HH diet reduced the FBW, WGR, SGR, FI, and FE of American eels (Figure 1A–E). However, both the HH+SAN100 and HH+SAN200 diets alleviated the HH-diet-induced decrease in FBW, WGR, SGR, FI, and FE (Figure 1A–E). SR was not significantly different among American eels fed these four experimental diets (Figure 1F). Thus, dietary sanguinarine supplementation could alleviate high-histamine-diet-induced inhibition in the growth performance and feed utilization of American eels.

### 3.2. Serum Lipid Indices

**Figure 2:**
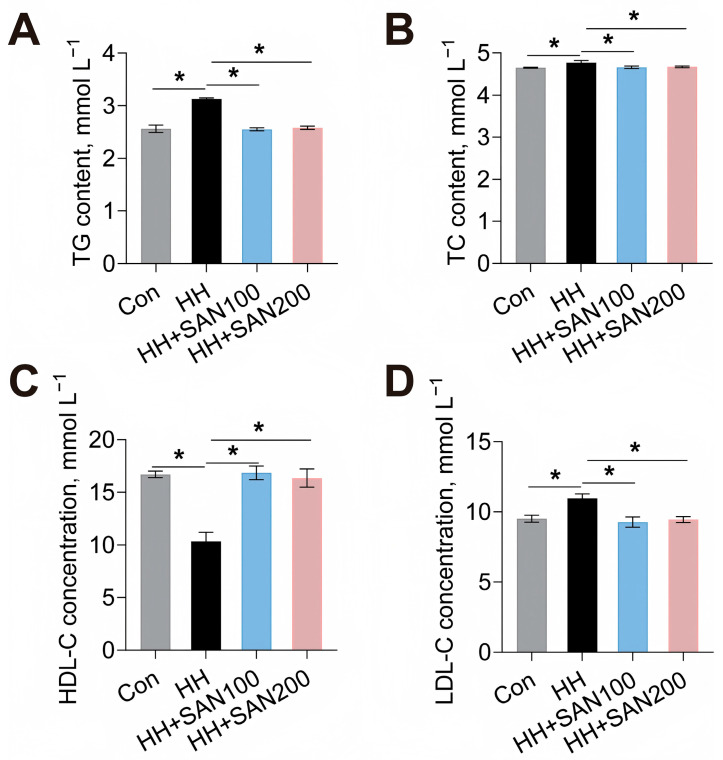
Effects of dietary histamine and sanguinarine on serum lipid parameters of juvenile American eels. (**A**) Serum TG content. (**B**) Serum TC content. (**C**) Serum HDL-C concentration. (**D**) Serum LDL-C concentration. Values are shown as mean ± S.D. (*n* = 4). Results were analyzed using independent *t*-test, * *p* < 0.05. Con, the control diet; HH, the high-histamine diet; HH+SAN100, the HH+SAN100 diet; HH+SAN200, the HH+SAN200 diet.

The effects of dietary sanguinarine and histamine on the serum lipid profile of American eels were then evaluated (Figure 2). The HH diet elevated the contents of serum TG, TC, and LDL-C while reducing serum HDL-C content compared to the control diet (Figure 2). In contrast, both the HH+SAN100 and HH+SAN200 diets lowered serum TG, TC, and LDL-C levels while increasing serum HDL-C content relative to the HH diet (Figure 2). Thus, dietary sanguinarine effectively alleviates high-histamine-diet-induced dysregulation of serum lipid metabolism in American eels.

### 3.3. Liver Histology

**Figure 3:**
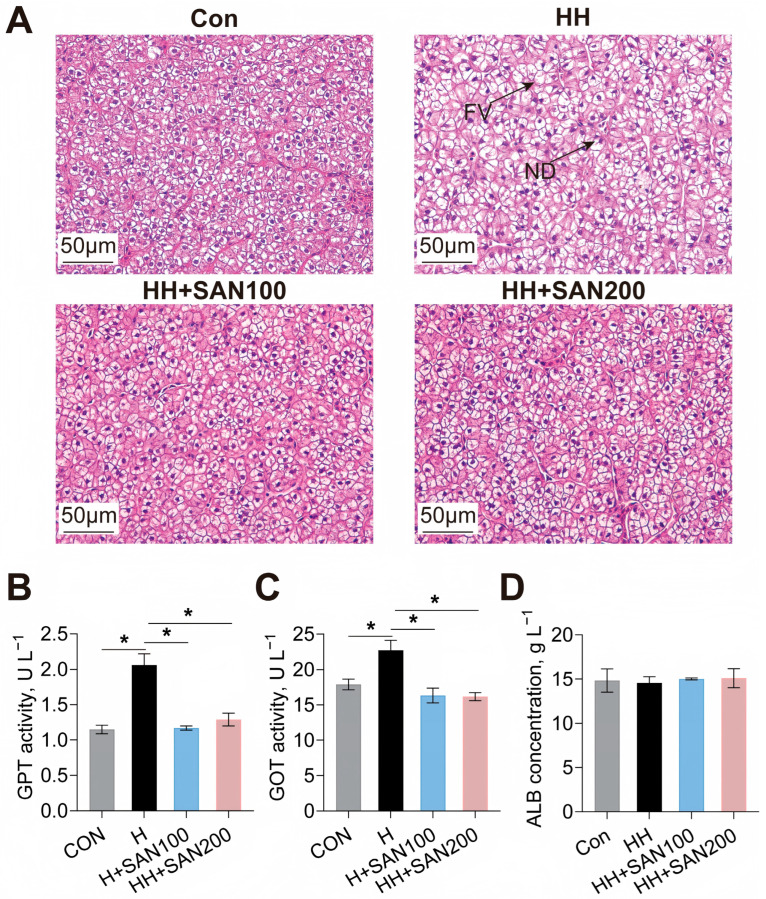
Effects of dietary histamine and sanguinarine on the hepatic structure of juvenile American eels. (**A**) Representative microphotograph of hematoxylin and eosin (H&E) staining, ND, nuclear deviation; FV, fat vacuoles. Scale bar, 50 μm. (**B**) Serum GPT activity. (**C**) Serum GOT activity. (**D**) Serum ALB concentration. Values are shown as mean ± S.D. (*n* = 4). Results were analyzed using independent *t*-test, * *p* < 0.05. Con, the control diet; HH, the high-histamine diet; HH+SAN100, the HH+SAN100 diet; HH+SAN200, the HH+SAN200 diet.

The effects of dietary sanguinarine and histamine on the hepatic morphology and serum liver function markers of American eels were assessed (Figure 3). Hepatocytes from American eels fed the control diet displayed distinct boundaries and centrally located spherical nuclei, whereas those fed the HH diet showed a high prevalence of hepatocyte vacuolar degeneration and nuclear displacement (Figure 3A). However, American eels fed the HH+SAN100 and HH+SAN200 diets displayed hepatic histomorphology comparable to those fed the control diet (Figure 3A). Consistently, the HH diet increased the activities of serum GPT and GOT compared to the control diet, while both the HH+SAN100 and HH+SAN200 diets reduced the activities of serum GPT and GOT compared to the HH diet (Figure 3B,C). Serum ALB activity did not differ significantly among the four dietary treatments (Figure 3D). Collectively, these structural and biochemical findings demonstrate that dietary sanguinarine supplementation effectively protects against high-histamine-diet-induced hepatocellular injury in American eels.

### 3.4. Hepatic Antioxidant Capacity

**Figure 4:**
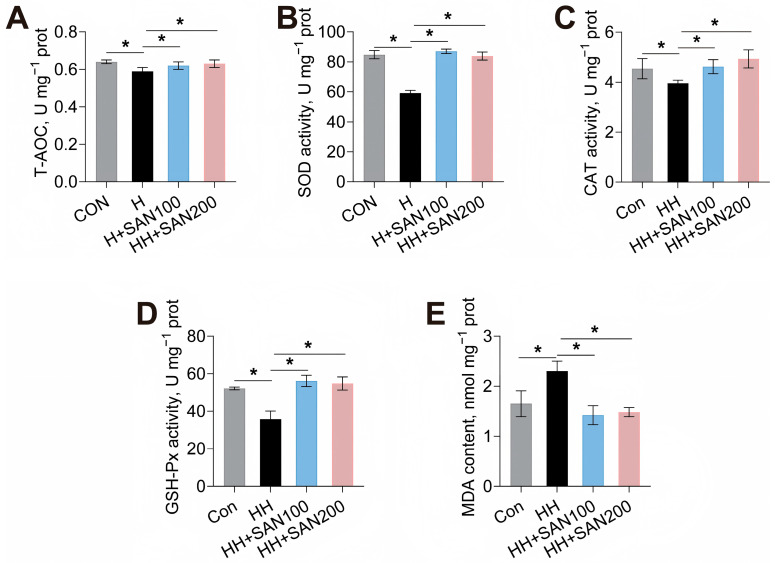
Effects of dietary histamine levels on the hepatic antioxidant capacity of juvenile American eels. (**A**) T-AOC. (**B**) SOD activity. (**C**) CAT activity. (**D**) GSH-Px activity. (**E**) MDA level. Values are shown as mean ± S.D. (*n* = 4). Results were analyzed using independent *t*-test, * *p* < 0.05. Con, the control diet; HH, the high-histamine diet; HH+SAN100, the HH+SAN100 diet; HH+SAN200, the HH+SAN200 diet.

The effects of dietary sanguinarine and histamine on hepatic antioxidant capacity in American eels were subsequently evaluated (Figure 4). Compared to the control diet, the HH diet significantly inhibited hepatic T-AOC, SOD, CAT, and GSH-Px activities, while concurrently increasing the MDA level (Figure 4). Conversely, both the HH+SAN100 and HH+SAN200 diets elevated hepatic T-AOC, SOD, CAT, and GSH-Px activities in comparison to the HH diet (Figure 4A–D). Moreover, American eels fed the HH+SAN100 and HH+SAN200 diets exhibited lower intestinal MDA levels than those receiving the HH diet (Figure 4E). Thus, dietary sanguinarine effectively mitigates high-histamine-diet-induced hepatic oxidative stress and lipid peroxidation in American eels.

### 3.5. Histological Assessment of Intestine

**Figure 5:**
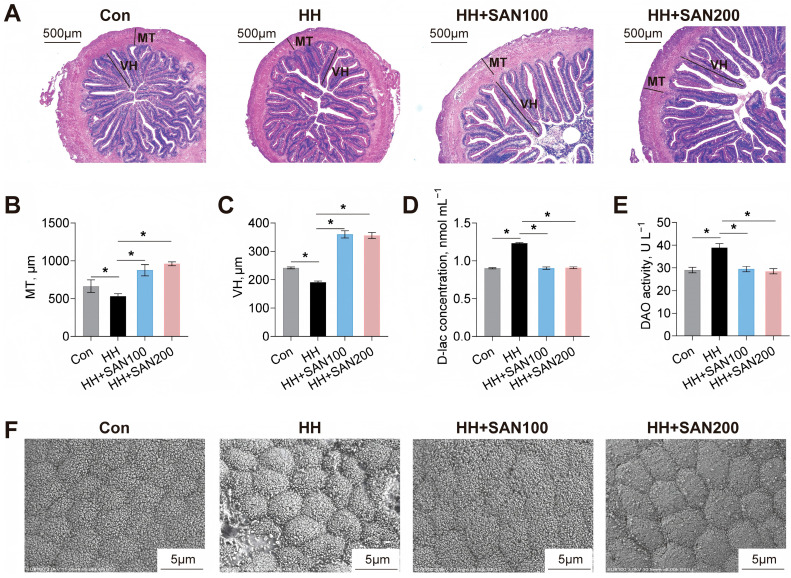
Effects of dietary histamine and sanguinarine on the intestinal structure of juvenile American eels. (**A**) Representative microphotograph of hematoxylin and eosin (H&E) staining. Scale bar, 500 μm. MT, muscular thickness. VH, villus height. (**B**,**C**) Statistical analysis of muscular thickness and villi height, related to Figure 3A. (**D**) Serum D-lac concentration. (**E**) Serum DAO activity. (**F**) Representative ultrastructural observation of the intestinal microvilli; scale bar = 5 μm. Values are shown as mean ± S.D. (*n* = 4). Results were analyzed using independent *t*-test, * *p* < 0.05. Con, the control diet; HH, the high-histamine diet; HH+SAN100, the HH+SAN100 diet; HH+SAN200, the HH+SAN200 diet.

To investigate the impacts of dietary sanguinarine and histamine on intestinal structure, H&E staining and SEM were utilized for morphological analysis (Figure 5). Compared with American eels fed the control diet, those fed the HH diet exhibited reduced villus height (VH) and muscular thickness (MT), accompanied by increased serum D-lac concentration and DAO activity (Figure 5A–E). Conversely, American eels receiving the HH+SAN100 and HH+SAN200 diets showed increased VH and MT, as well as decreased serum D-lac concentration and DAO activity, compared with those fed the HH diets (Figure 5A–E). Furthermore, SEM observed that the intestinal microvilli of American eels fed the control diet exhibited a dense and intact arrangement, whereas microvilli of American eels fed the HH diet were impaired (Figure 5F). Compared to the HH diet, both the HH+SAN100 and HH+SAN200 diets restored microvilli density and organization (Figure 5F). Collectively, these results suggest that dietary sanguinarine alleviates high-histamine-diet-induced impairment of intestinal mucosal integrity in American eels.

### 3.6. Intestinal Antioxidant Capacity

**Figure 6:**
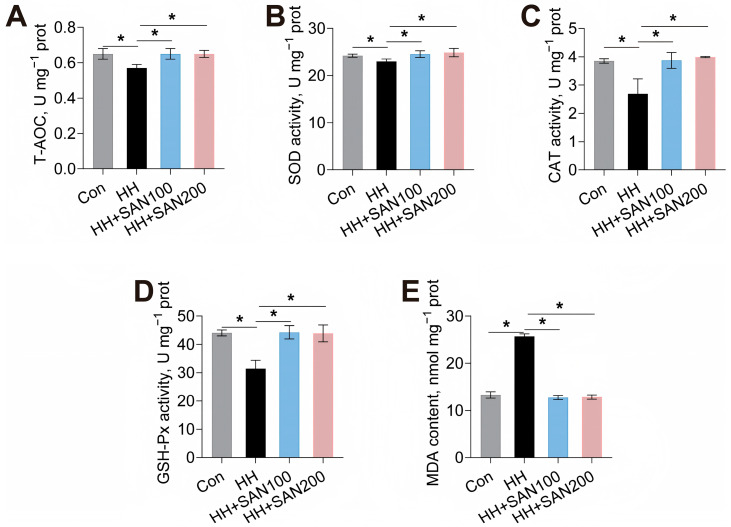
Effects of dietary histamine and sanguinarine on the intestinal antioxidant capacity of juvenile American eels. (**A**) T-AOC. (**B**) SOD activity. (**C**) CAT activity. (**D**) GSH-Px activity. (**E**) MDA level. Values are shown as mean ± S.D. (*n* = 4). Results were analyzed using independent *t*-test, * *p* < 0.05. Con, the control diet; HH, the high-histamine diet; HH+SAN100, the HH+SAN100 diet; HH+SAN200, the HH+SAN200 diet.

The effects of dietary sanguinarine and histamine on the intestinal antioxidant capacity of American eels are shown in Figure 6. The HH diet significantly decreased the activities of T-AOC, SOD, CAT, and GSH-Px and significantly increased the MDA level in the intestine compared with the control diet (Figure 6). Conversely, both the HH+SAN100 and HH+SAN200 diets elevated the intestinal activities of T-AOC, SOD, CAT, and GSH-Px in comparison to the HH diet (Figure 6A–D). Additionally, American eels fed the HH+SAN100 and HH+SAN200 diets exhibited lower intestinal MDA levels than those receiving the HH diet (Figure 6E). Thus, dietary sanguinarine alleviates high-histamine-diet-induced impairment of intestinal antioxidant capacity in American eels.

### 3.7. Intestinal Digestive Enzyme Activities

**Figure 7:**
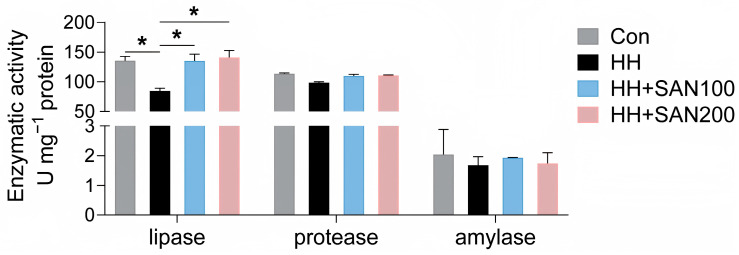
Effects of dietary histamine and sanguinarine on the activities of intestinal digestive enzymes in juvenile American eels. Values are shown as mean ± S.D. (*n* = 4). Results were analyzed using independent *t*-test, * *p* < 0.05. Con, the control diet; HH, the high-histamine diet; HH+SAN100, the HH+SAN100 diet; HH+SAN200, the HH+SAN200 diet.

As shown in Figure 7, the HH diet inhibited the activity of intestinal lipase relative to the control diet, while American eels fed the HH+SAN100 and HH+SAN200 diets exhibited higher intestinal lipase activity than those receiving the HH diet. Dietary sanguinarine and histamine had no effects on the activities of intestinal protease and amylase (Figure 7). Thus, dietary sanguinarine alleviates high-histamine-diet-induced inhibition of intestinal lipase activity in American eels.

### 3.8. Relative mRNA Level of Genes Associated with Intestinal Inflammation

**Figure 8:**
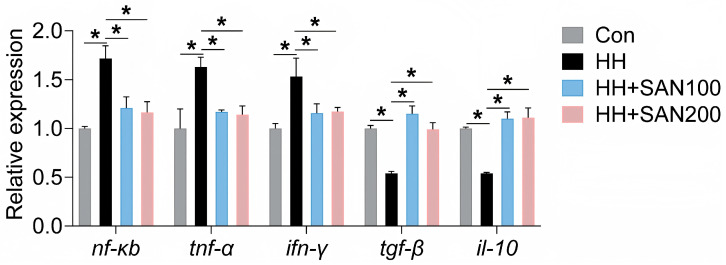
Effects of dietary histamine and sanguinarine on the relative expression levels of inflammatory-related genes in the intestine of juvenile American eels. Values are shown as mean ± S.D. (*n* = 4). Results were analyzed using independent *t*-test, * *p* < 0.05. Con, the control diet; HH, the high-histamine diet; HH+SAN100, the HH+SAN100 diet; HH+SAN200, the HH+SAN200 diet.

As presented in Figure 8, the HH diet increased the mRNA levels of *nf-κb*, *tnf-α*, and *ifn-γ*, while decreasing the mRNA levels of *tgf-β* and *il-10* compared to the control diet. American eels fed the HH+SAN100 and HH+SAN200 diets exhibited lower expressions of *nf-κb*, *tnf-α*, and *ifn-γ* but higher expressions of *tgf-β* and *il-10* than those receiving the HH diet (Figure 8). No notable variations were observed in the mRNA abundance of these intestinal inflammatory genes among American eels fed the control, the HH+SAN100, and the HH+SAN200 diets (Figure 8). Collectively, dietary sanguinarine alleviates the intestinal inflammatory response triggered by excessive dietary histamine.

### 3.9. Gut Microbiota Profiling

**Figure 9:**
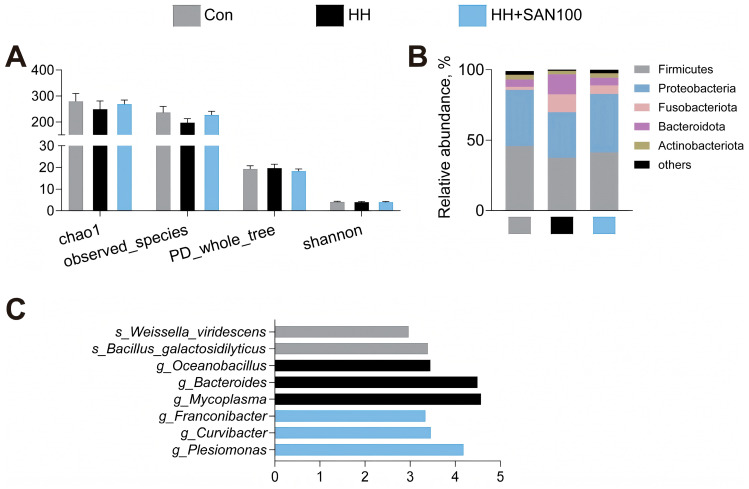
Effects of dietary histamine and sanguinarine on the alpha diversity (**A**), phylum (**B**), and genus (**C**) level of intestinal microbial of juvenile American eels in different treatment groups. Data are presented as means ± S.D. (*n* = 4). Con, the control diet; HH, the high-histamine diet; HH+SAN100, the HH+SAN100 diet.

In order to evaluate the impacts of dietary histamine and sanguinarine on the gut microbiotal composition of American eels, gut microbiota profiling was performed. Considering that dietary supplementation with 100 mg kg^−1^ and 200 mg kg^−1^ of sanguinarine showed no different effects on the intestinal structure, oxidant capacity, and inflammation of American eels, American eels that were fed the control, HH, and HH+SAN100 diets were subjected to the following analyzes. The results showed no significant changes in the Chao1 index, observed species, PD whole tree, and Shannon index among American eels fed the control, HH, and HH+SAN100 diets (Figure 9A). Additionally, we also observed that, at the phylum level, Firmicutes, Proteobacteria, Fusobacteria, Bacteroidetes, and Actinobacteriota were identified as the dominant bacterial groups in the intestines of American eels fed these experimental diets (Figure 9B). American eels fed the control diet and the HH+SAN100 diet exhibited a similar composition of gut microbiota at the phylum level, with a similar relative abundance of bacterial populations. Furthermore, the differential bacterial composition at the genus level in the intestine was analyzed (Figure 9C). American eels fed the control diet exhibited significantly higher relative abundances of *Bacillus galactosidilyticus* and *Weissella viridescens*, those fed the HH diet showed significantly higher relative abundances of *Mycoplasma*, *Bacteroides*, and *Oceanobacillus*, while those fed the HH+SAN100 diet displayed significantly higher relative abundances of *Plesiomonas*, *Curvibacter*, and *Franconibacter* (Figure 9C), indicating the significant differences in the relative abundances of specific bacteria among American eels fed these different diets.

### 3.10. Metabolomics Analysis of the Intestine

**Figure 10:**
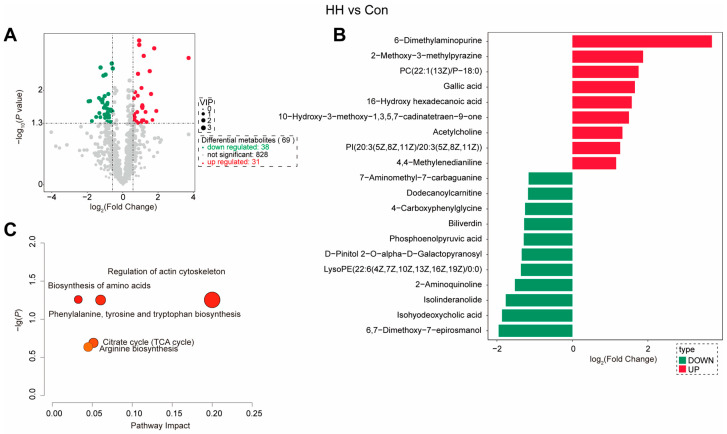
Metabolomics analysis of the intestine in juvenile American eels fed with histamine. (**A**) Differential metabolites (DMs) were visualized as a volcano plot map. (**B**) Differential metabolites (DMs) visualized as a histogram. (**C**) KEGG enrichment of DMs. Con, the control diet; HH, the high-histamine diet.

**Figure 11:**
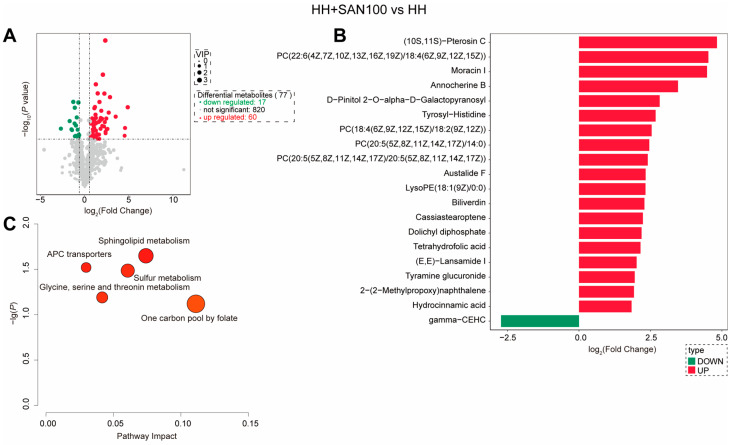
Metabolomics analysis of the intestine in juvenile American eels fed with histamine and sanguinarine. (**A**) Differential metabolites (DMs) were visualized as a volcano plot map. (**B**) Differential metabolites (DMs) visualized as a histogram. (**C**) KEGG enrichment of DMs. HH, the high-histamine diet; HH+SAN100, the HH+SAN100 diet.

Intestinal metabolomic profiling was performed to characterize metabolic alterations associated with dietary histamine and sanguinarine in American eels fed the control, HH, and HH+SAN100 diets. Compared to American eels fed the control diet, 69 differential metabolites (DMs) were identified in those that received the HH diet, including 31 upregulated and 38 downregulated metabolites (Figure 10A,B). Furthermore, KEGG pathway enrichment analysis of these DMs were significantly associated with the regulation of actin cytoskeleton, biosynthesis of amino acids, phenylalanine, tyrosine and tryptophan (Phe/Tyr/Trp) biosynthesis, citrate cycle, and arginine biosynthesis (Figure 10C). Additionally, compared to American eels fed the HH diet, 87 differential DMs were detected in those that received the HH+SAN100 diet (Figure 11A). Among these DMs, 31 metabolites were upregulated and 38 metabolites were downregulated following dietary sanguinarine supplementation (Figure 11A,B). KEGG pathway enrichment analysis showed that these DMs were enriched in sphingolipid metabolism, sulfur metabolism, APC transporters, glycine, serine and threonine (Gly/Ser/Thr) metabolism, and one-carbon pool by folate were significantly affected (Figure 11C). Overall, these results suggest that dietary sanguinarine supplementation partially reshapes high-histamine-diet-disrupted intestinal metabolism, particularly pathways linked to amino-acid metabolism, energy metabolism, and membrane-associated processes.

## 4. Discussion

Growth performance and feed utilization serve as the macroscopic indicators of nutrient assimilation and overall physiological health in fish [3,20]. In the present study, excessive dietary histamine markedly impaired growth performance and feed utilization in American eels, as evidenced by reductions in FBW, WGR, SGR, FI, and FE. This high-histamine-diet-induced growth retardation aligns with previous reports in American eels and other teleosts, including grouper and striped catfish [3,18,21], which demonstrated that excessive dietary histamine acts as a potent anti-nutritional and toxicological factor for some fish species. Notably, our findings revealed that dietary sanguinarine supplementation effectively alleviated this high-histamine-diet-induced growth inhibition and reduced feed utilization. Sanguinarine is a bioactive alkaloid that has been reported to promote growth and improve feed conversion efficiency [14,22]. Moreover, dietary sanguinarine supplementation has been shown to attenuate growth impairment in grass carp fed high levels of cottonseed meal and rapeseed meal, suggesting it possesses broader stress-mitigating properties [14]. Consequently, these observations highlight the efficacy of sanguinarine as a nutritional intervention to mitigate high-histamine-diet-induced growth retardation and reduced feed utilization in American eels.

The liver governs systemic metabolic homeostasis, while the intestine serves as the primary site for nutrient absorption and barrier defense [19,20]. Both liver and intestine are commonly prime targets for dietary toxicants [2,23]. The present results showed that sanguinarine exerted a protective effect against high-histamine-diet-induced injury in both the liver and intestine of American eels. The structural integrity of the liver and intestine is fundamental for nutrient assimilation and systemic metabolic homeostasis [19,24]. American eels fed the high-histamine diet displayed typical signs of hepatocellular damage, including vacuolar degeneration, nuclear displacement, and elevated serum GPT and GOT activities, whereas these alterations were markedly alleviated by dietary sanguinarine supplementation. These findings suggest that the supplementation of dietary sanguinarine addition contributed to the preservation of hepatocyte integrity and membrane stability under histamine stress [25,26]. Similar protective effects of sanguinarine or sanguinarine-containing plant extracts on hepatocyte integrity have been reported under other stress conditions, including carbon tetrachloride, heat, and hydrogen peroxide [13,27,28]. Concurrently, in the intestine, the high-histamine diet reduced villus height and muscular thickness, disrupted microvilli organization, and increased serum DAO activity and D-lac concentration, demonstrating impairment of the mucosal structure and barrier integrity [19]. Consistent with its hepatoprotective role, dietary supplementation with sanguinarine markedly alleviated the high-histamine-diet-induced structural abnormalities in the intestine. This aligns with previous findings in grass carp, where dietary sanguinarine improved intestinal structural integrity under the stress of cottonseed meal, rapeseed meal, and high-fat diets [14,15]. Collectively, the concurrent restoration of structural integrity of the liver and intestine highlights that sanguinarine provides structural defense against histamine toxicity.

Previous studies implied that oxidative stress and lipid peroxidation serve as the important links to dietary-histamine-induced structural damage in the intestine and liver [7,15,18,21]. In the present study, the high-histamine diet decreased T-AOC and the activities of major antioxidant enzymes, including SOD, CAT, and GSH-Px, in both the liver and intestine, indicating compromised antioxidant defenses [29,30,31]. Reduced antioxidant capacity is inevitably unable to inhibit the accumulation of reactive oxygen species [29]. Consequently, these free radicals attack the polyunsaturated fatty acids within cellular phospholipid bilayers, initiating severe lipid peroxidation [30]. The dramatic elevation of MDA levels in both the intestine and liver provides direct biochemical evidence of this oxidative damage [30]. It is this lipid peroxidation that destroys membrane fluidity and integrity, serving as the fundamental biochemical cause for the observed intestinal microvillar collapse, hepatocellular vacuolization, and the subsequent leakage of intracellular GOT, GPT, D-lac, and DAO into the bloodstream [29,30]. In contrast, dietary sanguinarine supplementation restored T-AOC and antioxidant enzyme activities and reduced MDA accumulation in both tissues. This antioxidant intervention preserved the phospholipid bilayers of enterocytes and hepatocytes, thereby restoring dense intestinal microvilli and distinct hepatocyte boundaries [29,31]. Consistent with our findings, Shi et al. [13] reported that dietary sanguinarine increased hepatic antioxidant capacity and alleviated liver injury in rice field eel under hydrogen peroxide stress. Similarly, Liu et al. [14] showed that dietary sanguinarine reduced MDA accumulation and mitigated intestinal injury in grass carp fed diets with high levels of cottonseed and rapeseed meals. Overall, these integrated structural and oxidative responses support the finding that the protective effects of dietary sanguinarine on intestinal and hepatic structure were closely accompanied by improvements in antioxidant status.

Serum lipid profiles, including TG, TC, HDL-C, and LDL-C, are widely used biomarkers of systemic lipid homeostasis [32]. In the present study, excessive dietary histamine disrupts systemic lipid homeostasis in American eels, characterized by increased TG, TC, and LDL-C and decreased HDL-C, suggesting impaired lipid transport and clearance under high-histamine diet [32]. Similarity, Cai et al. [33] reported that excessive dietary histamine caused hyperlipidemia in American eels. This disturbance may be partly by compromised hepatic health under the dietary histamine stress, as the liver is central to lipid synthesis, lipoprotein assembly, and lipid clearance [34]. The excessive dietary-histamine-induced hepatic injury may have substantially impaired the hepatic capacity for proper lipid packaging and clearance. Remarkably, dietary sanguinarine supplementation normalized this dyslipidemic state induced by the high-histamine diet. By alleviating hepatic oxidative stress and preserving hepatocellular architecture, dietary sanguinarine supplementation appears to facilitate the recovery of the hepatic functional competence to regulate lipoprotein secretion [34,35]. This hypolipidemic and hepatoprotective effect aligns with our recent research demonstrating that dietary sanguinarine supplementation concurrently increased serum HDL-C level and improved hepatic structural integrity [35]. Ultimately, the restoration of serum lipid homeostasis further corroborates that dietary sanguinarine rescues American eels from lipid metabolic disorders by securing hepatic structural and functional integrity against high-histamine-diet-induced toxicity.

The intestine is central to nutrient digestion and absorption, and these functions are tightly linked to intestinal structural integrity [18,36]. Accordingly, dietary-histamine-induced impairment of intestine may translate into reduced digestive capacity. Our results showed that the high-histamine diet significantly inhibited intestinal lipase activity, which consistent with the findings in our previous study [3,18]. This functional deficit likely relates to the mucosal structural damage described earlier, because disruption of microvilli can directly diminish the effective digestive and absorptive interface and potentially impair the local conditions required for optimal lipase activity [36]. In contrast, dietary sanguinarine supplementation restored intestinal lipase activity relative to the high-histamine diet, which is consistent with its concurrent improvement of villus development and microvilli density mentioned above. Together, these results indicate that the recovery of lipase activity likely reflects a restoration of intestinal mucosal integrity and function by sanguinarine under high-histamine stress.

The intestinal mucosa represents the largest immunological interface in teleosts, and homeostasis depends on maintaining an appropriate balance between pro- and anti-inflammatory signaling [37,38]. NF-κB is a central transcriptional regulator of inflammatory signaling, and TNF-α and IFN-γ are representative pro-inflammatory cytokines, whereas TGF-β and IL-10 are commonly regarded as anti-inflammatory mediators [38]. In this study, excessive dietary histamine induced a pro-inflammatory response in the intestine, as evidenced by increased *nf-κb*, *tnf-α*, and *ifn-*γ expression and decreased *tgf-β* and *il-10* expression. This inflammatory response likely exacerbated the intestinal mucosal injury, as pro-inflammatory cytokines can disrupt tight junction and induce enterocyte apoptosis [39]. Notably, dietary sanguinarine supplementation effectively reversed the high-histamine-diet-triggered pro-inflammatory response. Sanguinarine has been recognized as a potent inhibitor of the NF-κB signaling pathway, which is responsible for its anti-inflammatory effects [12]. Similarity, Liu et al. [14] also uncovered that the addition of dietary sanguinarine in the diet effectively downregulated the expression of *tnf-α* in the intestine of grass carp fed cottonseed and rapeseed meal diets. Therefore, our results indicated that dietary sanguinarine supplementation alleviated intestinal inflammation caused by a high-histamine diet in American eels.

The gut microbiota plays a pivotal role in regulating nutrient metabolism, modulating immune responses, and maintaining the integrity of the intestinal barrier [40]. In the present study, although alpha diversity indices did not differ among all groups, the microbial community composition was reconstructed. Lefse analysis revealed that the relative abundances of *Mycoplasma*, *Bacteroides*, and *Oceanobacillus* were higher in American eels fed the HH diet in comparison with those fed the control and HH+SAN100 diets. Most *Mycoplasma* species are pathogenic and can induce cytotoxicity through the release of hydrogen peroxide and exotoxins [41]. Previous studies have associated *Mycoplasma* with intestinal inflammation in largemouth bass (*Micropterus salmoides*) [41]. Increased *Bacteroides* abundance has also been observed in grass carp fed diets containing high-level cottonseed and rapeseed meal, and prior research has identified a positive correlation between *Bacteroides* levels and the expression of *tnf-α* [14,42]. *Oceanobacillus* has been detected as a commensal taxon in fish skin and intestine, but its pathogenic potential remains unclear [43]. Therefore, excessive dietary histamine promoted the proliferation of potentially harmful bacteria in the intestine of American eels. Additionally, our findings demonstrated that the supplementation of sanguinarine in the diet effectively suppressed the high-histamine-induced stimulated growth of *Mycoplasma* and *Bacteroides*. In comparison with the control and HH diets, the HH+SAN100 diet increased the relative abundance of *Plesiomonas*, *Curvibacter*, and *Franconibacter* in the intestine. *Plesiomonas* is generally considered a conditional pathogen, but its pathogenicity is closely related to bacterial abundance, host immune status, and environmental conditions [44]. At low abundance, *Plesiomonas* may exist as a commensal component of the environmental microbiota in healthy hosts [45]. *Curvibacter* has been found at higher levels in healthy fish intestines compared to those with intestinal damage, suggesting potential probiotic effects [46,47]. As the specific functions of *Curvibacter* and *Franconibacter* in fish remain poorly characterized, further experimental evidence is required to confirm their roles. Collectively, these results indicate that dietary sanguinarine supplementation effectively mitigates high-histamine-induced intestinal dysbacteriosis by inhibiting the proliferation of deleterious taxa and fostering a more balanced microbial ecosystem.

To further assess the effects of dietary histamine and sanguinarine on intestinal physiological status, metabolomics analysis was carried out to determine intestinal metabolites. In the present study, excessive dietary histamine increased intestinal acetylcholine levels in American eels, indicating significant activation of actin cytoskeleton regulation under the high-histamine diet. Under stress conditions, activation of the actin cytoskeletal pathway is commonly associated with cell contraction, tight-junction rearrangement, and altered epithelial permeability as a response to external stimuli [48,49]. These results therefore suggest that excessive dietary histamine may trigger cytoskeletal remodeling in intestinal epithelial cells, which likely serves as the molecular driver for the observed intestinal histomorphological changes induced by high-histamine diet. Furthermore, excessive dietary histamine led to the downregulation of intestinal L-arginine, reflecting suppression of the arginine biosynthesis pathway. Arginine is an essential amino acid and plays a pivotal role in maintaining intestinal homeostasis by promoting mucosal repair and improving structural integrity [50]. Moreover, key metabolites within arginine biosynthesis pathway, such as ornithine and glutamine, are recognized for their roles in mitigating inflammatory responses and oxidative damage [51,52]. Additionally, excessive dietary histamine decreased levels of phosphoenolpyruvate and phenylpyruvate in the intestine, suggesting inhibition of Phe/Tyr/Trp biosynthesis and the citrate cycle. Phosphoenolpyruvate, a critical intermediate in both glycolysis and gluconeogenesis, serves as a primary precursor for the biosynthesis of aromatic amino acids, while phenylpyruvate is a key intermediate in the metabolic pathways of phenylalanine and tyrosine [53,54]. A decrease in these metabolites may, on the one hand, directly constrain the biosynthesis of phenylalanine, tyrosine, and tryptophan, thereby potentially affecting protein synthesis and antioxidant capacity, which could ultimately disturb intestinal homeostasis [55]. On the other hand, the decreased abundance of phosphoenolpyruvate may reduce citrate cycle intermediates, thereby constraining the substrate supply for this cycle, which inevitably leads to a decline in energy metabolism efficiency and impairing the maintenance of normal physiological functions in intestinal epithelial cells [56]. Collectively, these intestinal metabolomics alternation provide a molecular basis for the reduced antioxidant capacity and structural impairment observed in American eels fed with a high histamine level.

Dietary sanguinarine supplementation significantly affected sphingolipid metabolism, Gly/Ser/Thr metabolism, one-carbon pool by folate, sulfur metabolism, and APC transporters. Our findings revealed that dietary sanguinarine supplementation significantly reduced the level of intestinal sphingosine, a catabolic product of sphingolipids, which exhibited anti-inflammatory effects at low concentrations [57]. The reduction in sphingosine reflects attenuated sphingolipid catabolism. Given that serine is a critical precursor for sphingolipid biosynthesis, the decelerated sphingolipid catabolism may have altered serine utilization, potentially contributing to its observed accumulation [58]. Previous studies have shown that serine can enhance the antioxidant capacity and stress resistance of fish [59]. Furthermore, Gly/Ser/Thr are interconverted through tetrahydrofolate-dependent enzymatic systems, and the concurrent elevation of tetrahydrofolate and serine suggests enhanced Gly/Ser/Thr metabolism coupled with one-carbon unit transfer [60]. The active one-carbon units generated through this pathway are essential for cellular biosynthesis and maintain metabolic homeostasis and facilitate the repair of the damaged intestinal mucosa [61]. Additionally, the increases in serine and methanesulfonate levels reflect an activation of sulfur metabolism. As the fundamental source for sulfur-containing amino acids, sulfur metabolism is integral to glutathione biosynthesis and crucial for bolstering antioxidant defenses [62]. The enrichment of the APC transporter pathway indicates a modulation of transmembrane transport efficiency, which plays a vital role in protecting against dietary toxicants and reactive oxygen species in the intestine [63]. Collectively, the dietary-sanguinarine-supplementation-induced alternations in the one-carbon pool by folate, Gly/Ser/Thr metabolism, sphingolipid metabolism, sulfur metabolism, and ABC transporter pathways provide a metabolomic basis for its efficacy in mitigating oxidative injury and restoring intestinal structural integrity under high-histamine-diet stress.

Although the present study demonstrated that dietary sanguinarine supplementation could alleviate excessive dietary-histamine-induced intestinal and hepatic damage in American eels, several limitations should be acknowledged. First, the 10-week feeding trial, while sufficient to observe notable metabolic, physiological, and morphological changes, represents a relatively short duration in the context of the entire aquaculture cycle of American eels. Consequently, the long-term effects of dietary sanguinarine and histamine on the growth performance and chronic health status of American eels remain to be fully elucidated. Second, the present experimental design did not include a sanguinarine-only treatment group. Although the current results clearly demonstrate the ameliorative effects of sanguinarine against histamine-induced damage, the absence of a sanguinarine-only group limits our ability to evaluate the baseline physiological impacts and potential long-term safety of sanguinarine when administered alone to healthy fish. Therefore, future studies involving extended feeding periods and comprehensive experimental designs, including a sanguinarine-only group, are warranted to validate our broader conclusions and to optimize the application of sanguinarine in eel aquaculture.

## 5. Conclusions

In summary, this study demonstrated that dietary sanguinarine supplementation at 100–200 mg kg^−1^ effectively mitigated the adverse effects of a high-histamine diet on the growth performance, feed utilization, serum lipid homeostasis, hepatic and intestinal structure and antioxidant defense, and intestinal inflammation in American eels. Microbiota profiling and intestinal metabolomic analyses further indicated that supplementation with 100 mg kg^−1^ of sanguinarine in the diet also suppressed the proliferation of harmful bacteria and modulated intestinal metabolic profiles caused by the high-histamine diet. Overall, these findings show that sanguinarine represents a practical additive to reduce high-histamine-diet-induced health risks and may facilitate the safer use of brown fishmeal in the feeds of American eels and other histamine-sensitive fish species.

## Figures and Tables

**Table 1:** Formulation and proximate composition analysis (% of dry weight basis) of the basal diet.

Ingredients, %	
White fishmeal	32.6
Brown fishmeal	35
α-starch	25
Expanded soybeans	2
Yeast powder	2
Choline chloride	0.5
Betaine	1
Ca(H_2_PO_4_)_2_	0.5
Mineral premix ^a^	1
Vitamin premix ^b^	0.4
Total	100
Proximate analysis %, dry weight	
Crude protein	47.87
Crude lipid	5.17
Crude ash	12.98
Moisture	6.22

^a^ Minerals premix (mg kg^−1^ diet): CuCl_2_ 12.05, FeCl_2_·H_2_O 666.67, MnSO_4_·H_2_O 94.34, ZnSO_4_·H_2_O 202.89, Ca(IO_3_)_2_ 2.59, Na_2_SeO_3_ 0.89, CoSO_4_ 3.64. ^b^ Vitamin premix (IU or mg kg^−1^ diet): Vitamin A 14,000.00 IU, Vitamin C 200.00 mg, Vitamin D 3200.00 IU, Vitamin E 40.00 mg, Vitamin K 16.00 mg, Vitamin B1 20.00 mg, Vitamin B6 16.00 mg, folic acid 4.80 mg, nicotinic acid 80.00 mg.

**Table 2:** The primers for real-time PCR analysis.

Genes	Forward Primer (5′–3′)	Reverse Primer (5′–3′)	Accession No.
*tnf-α*	CCAGACCAGAGCCAAGAAGG	AGGTATGGCCCGTGTCTTTG	MT861110
*ifn-γ*	AATGACAAATGACGTGAATAGG	TTCAGCATGTCCGACAGG	MT861111
*il-10*	CCAGAGACGACCTGTTGCTT	AACGCGTCATCTCCCCATTT	XM064306058
*tgf-β*	CTGGTGTTCTGGGAAATCGC	ATGACCCTCAGCGCCTCAC	XM064301364
*nf-κb*	GAGCAACGACACCACCAAGA	TGCTTACAGTCCTTGCCGAC	XM064335268
*gapdh*	ATTGGTCGTCTTGTGACCCG	GTCCGTGGGTGGAGTCATAC	XM064310421

Abbreviations: *gapdh*, glyceraldehyde-3-phosphate dehydrogenase; *ifn-γ*, interferon gamma; *il-10*, interleukin 10; *nf-κb*, nuclear factor kappa-b; *tgf-β*, transforming growth factor beta; *tnf-α*, tumor necrosis factor alpha.

## Data Availability

The data that support the findings of this study are available from the corresponding author upon reasonable request.

## Abbreviations

The following abbreviations are used in this manuscript:

CAT	catalase
Con	control diet
DAO	diamine oxidase
D-lac	D-lactate
FBW	final body weight
FE	feed efficiency
FI	feed intake
gapdh	glyceraldehyde-3-phosphate dehydrogenase
GOT	glutamic-oxalacetic transaminase
GPT	glutamic-pyruvic transaminase
GSH-Px	glutathione peroxidase
HDL-C	high-density lipoprotein cholesterol
HH	high-histamine diet
H&E	hematoxylin and eosin
IBW	initial body weight
ifn-γ	interferon gamma
il-10	interleukin 10
LDL-C	low-density lipoprotein cholesterol
MDA	malondialdehyde
MT	muscular thickness
nf-κb	nuclear factor kappa-b
RT-PCR	real-time quantitative PCR
SAN	sanguinarine
SEM	scanning electron microscope
SOD	superoxide dismutase
SGR	specific growth rate
SR	survival rate
S.D.	standard deviation
TC	total cholesterol
TG	triglyceride
tgf-β	transforming growth factor beta
tnf-α	tumor necrosis factor alpha
T-AOC	total antioxidant capacity
VH	villus height
WGR	weight gain rate
